# A Manual of the Diseases of the Skin

**Published:** 1886-06

**Authors:** 


**A Manual of the Diseases of the Skin; their Description, Diagnosis and Treatment.** By BALMANNO SQUIRE, M. B., London, Surgeon to the British Hospital for Diseases of the Skin, London, Eng. 16mo. Cloth. 194 pages. Price, $1. Chicago: A. N. Marquis & Co.

This is an excellent little work which has gone through four editions in England. The special characteristics of the work are simplicity in the division and grouping of the subject matter so that the reader is able to turn at once to any particular disease or class of diseases respecting which he may desire information. It is one of the best of the smaller hand-books on the subject.

